# The sensory evaluation and antimicrobial efficacy of *Lactobacillus acidophilus* supernatant on *Salmonella enteritidis* in milk

**DOI:** 10.1002/fsn3.3883

**Published:** 2023-12-19

**Authors:** Abbas Kamali, Hedayat Hosseini, Razzagh Mahmoudi, Babak Pakbin, Nematollah Gheibi, Amir Mohammad Mortazavian, Saeideh Shojaei

**Affiliations:** ^1^ Department of Food Science and Technology, Faculty of Nutrition Sciences and Food Technology, National Nutrition and Food Technology Research Institute Shahid Beheshti University of Medical Sciences Tehran Iran; ^2^ Medical Microbiology Research Center Qazvin University of Medical Sciences Qazvin Iran; ^3^ Department of Chemistry, Werner Siemens Chair of Synthetic Biotechnology Technical University of Munich (TUM) Garching bei München Germany; ^4^ Cellular and Molecular Research Center Qazvin University of Medical Sciences Qazvin Iran

**Keywords:** fluctuating temperatures, *Lactobacillus acidophilus*, milk, *Salmonella enteritidis*, sensory evaluation, supernatant

## Abstract

Postbiotics are metabolites derived from living probiotic bacteria like Lactobacillus strains, during the fermentation process and/or produced in pure form on laboratory scales. These compounds, depending on the type of probiotic from which they are prepared, have specific antibacterial agents such as: organic acids, bacteriocins, short‐chain fatty acids, and peptides. The objective of this study was to investigate the effect of *Lactobacillus acidophilus* supernatant (LAS) on the growth pattern of *Salmonella enteritidis* at fluctuating temperatures and the sensory evaluation of milk that contains this probiotic. Baranyi and Roberts's model determined the best‐fit curve for the microbial growth. According to mathematical equations, the highest and lowest specific growth (*μ*_max_) rates of *S. enteritidis* were obtained at 0.055 h^−1^ and 0.0059 h^−1^ and also highest and lowest maximum generation time (MGT) values were obtained at 20.06 h and 8.85 h, respectively. Sensory evaluation by the Triangel test reveals that LAS could not establish a significant (*p* > .05) adverse effect on milk perceptible. Regarding the results obtained in the present study, LAS, without causing adverse sensory change, could act as a safe food additive for the control of bacterial pathogens and reducing food waste, particularly in milk and milk‐containing food products.

## Graphical abstract

The objective of this study was to investigate the effect of L. acidophilus supernatant (LAS) on the growth pattern of *S. entritidis* at fluctuating temperatures and the sensory evaluation of milk that contains this probiotic. Regarding the results obtained in the present study, LAS, without causing adverse sensory changes, could act as a safe food additive for the control of bacterial pathogens and reduction in food waste, particularly in milk and milk‐containing food products.

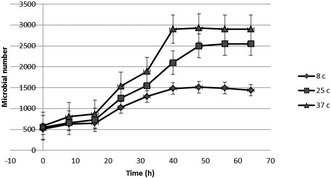

## 1 INTRODUCTION

Probiotics is a phrase in the Greek language, denoting life. Probiotics, in a scientific context, are known as substances or microorganisms that enhance the health of the host (Krawczyk & Banaszkiewicz, 2021). According to the definitions of WHO and FAO in 2020, probiotics, known as live microorganisms, when administered in adequate amounts, confer a health benefit on the host (Morelli & Capurso, 2012). A high variety of traditional fermented dairy products, such as curd, yogurt, cheese, colostrum, fermented milk beverage, Shiraz (produced in the south of Iran), and Tarkhineh (produced in the west of Iran), are the main sources of beneficial probiotic strains in different countries. These products are a rich source of a variety of types of probiotic bacteria (Haghshenas et al., 2021; Kiani et al., 2021). Most microorganisms that are recognized as probiotics are Gram‐positive, non‐flagellated, non‐spore‐forming, microaerophilic, and rods or coccobacillus, and the most important of them are *Lactobacillus* and *Bifidobacterium* commonly used in probiotic products. Probiotics are also made up of good yeast strains belonging to the genera *Saccharomyces cerevisia* var. *boulardi* and *Kluyveromyces marxianus* (e.g., *Saccharomyces boulardii*). Probiotic products in three general categories, including food products, nutritional supplements, and drugs, may contain one or more selected microbial strains, such as *Lactobacillus*, *bifidobacterium*, *enterococcus*, *streptococcus*, and *lactococcus*. Postbiotics, as safe surrogate groups of probiotics, are defined as viable and unviable probiotic metabolites such as cell‐free supernatant (CFS), metabolic waste, or biological compounds from probiotic activity, including hydroperoxide, organic acids, acetaldehydes, ethanol, diacetyl, bacteriocin, and short‐chain fatty acids that offer health effects for the host (Garrote et al., 2015). Therefore, a large number of probiotic benefits have also been described for postbiotics. Recent studies show that these metabolites have a broad inhibitory property toward the human pathogenic agent and therefore can be used, as an alternative to antibiotics.

*Lactobacillus acidophilus*, as a probiotic bacterium, can be added to many food fermentation processes and preserve the products by producing bacteriocins and lactic acid (Çakmakçi et al., 2012). Therefore, postbiotic products of *L. acidophilus*, including metabolites, supernatant, and cell‐free extract, are highly recommended to control the growth of pathogenic bacteria in food products. This probiotic is a fastidious organism and needs a variety of nutrients to grow, and because of this, it is generally not used alone for fermentation (Aller et al., 2014). For this reason, the use of the supernatant of this bacterium can be proposed as a substitute for live bacteria in the food industry.

Milk, as a nutritive drink, contains a variety of short‐chain fatty acids, protein, vitamins, minerals, and carbohydrates, which have vast consumption among all age groups (Sundarraj et al., 2018). The presence of such nutrients, in addition to providing the nutritional needs of consumers, can also create a suitable growth medium for a wide range of disease‐causing and spoilage bacteria. A wide variety of microorganisms, including Gram‐negative and Gram‐positive bacteria and a broad range of fungal, can spoil milk and dairy products (Metchnikoff, 1996). Although the use of food‐conserving methods such as pasteurization, sterilization, antimicrobial packaging, and dying always increases their storage period, it is inevitable to reduce the nutritional value and create an inappropriate appearance and unpleasant taste (Delgado et al., 2006). For this reason, the use of new food preservation methods is inevitable, and in the meantime, probiotics and postbiotics have been considered as innovative methods. Several studies have verified the effectiveness of probiotic strains in inhibiting the growth of spoilage microorganisms in food products.

The principles of sensory evaluation are of great importance for preference. Undoubtedly, commercial success cannot be achieved without considering the sensory attributes of products, and this principle has been proven to all consumers, food producers, and retailers. The organoleptic characteristics of milk, such as sight, smell, and taste, are fundamental to the daily industry (Kaur et al., 2014). Because of this, performing a sensory evaluation of the product with a new formulation or process before entering the market will be very important.

*Salmonella* spp., as a Gram‐negative microorganism and facultative anaerobic bacteria belonging to the family *Enterobacteriaceas*, is one of the most common pathogens that cause foodborne infection and mortality globally by transmitting to humans through raw food animal products such as: poultry meat and dairy products (Yan et al., 2004). Among the 2500 *salmonella* serotypes, *S. enteritidis*, *S. typhimurium*, *S. hadar*, and *S. infantis*, were identified as major public health concerns (Omar et al., 2018). *Salmonella* spp. that causes infection in cattle has been reported in various countries, and among them, *Salmonella enteritidis* is more prevalent in cattle.

Among the main causes of food spoilage that include microbial growth, chemical reactions, and physical damage, microbial activity is the most essential factor. There are different methods to prevent food spoilage and increase its shelf life, e.g, chilling, freezing, freeze‐drying, heating, sugar‐addition, salting, drying, preservatives, fermentation, smoking or oxygen removal, acidification, and canning (Leistner & Gorris, 1994). Adding probiotics to food products is a novel concept that can play a protective role against pathogens and improve shelf life during storage.

The aim of this study is to survey the inhibition of *S. enteritidis* growth in proximity to freeze‐dried *Lactobacillus acidophilus* supernatant (LAS) at different incubation temperatures. The purposes of the present study were (i) to determine the Minimum Inhibitory Concentration (MIC) of *L. acidophilus* against *S. enteritidis* in medium culture, (ii) prediction of microbial growth of *S. enteritidis* in milk exposed to MIC and sub‐MIC of freeze‐dried LAS in different temperatures (8, 25, and 37°C) by determination growth curve, and (iii) sensory evaluation of treated milk by freeze‐dried LAS.

## 2 MATERIALS AND METHODS

### 2.1 Bacterial strains, media, and growth condition

Lyophilized probiotic *L. acidophilus* strain PTCC 1932 was ordered and purchased from the Iranian Biological Resource center. This probiotic was grown in Tryptic Soy Broth (TSB, ProMedia) medium at 37°C for 24 h. To obtain net culture, aliquots (0.1 mL) of the activated culture were transferred to Tryptic Soy Agar (TSA, ProMedia) medium at 37°C for 48 h, then the colony was picked up and inoculated in TSB at 37°C for 18 h. *S. enteritidis* ATCC 13076, as a pathogen strain, was used during the experiment. The colony of stock culture was activated by incubation into Brain Heat Infusion broth (BHI, ProMedia) medium and incubation at 37°C for 24 h.

### 2.2 Preparation of LAS and freeze‐drying

LAS was obtained from centrifuging activated net culture in TSB at 3000 rate per minute (RPM) for 10 min and then 12,000 RPM for 15 min. After PH determination (glass electrode), the supernatant was collected and passed through a sterilized 0.22 μ‐pore size filter (Khiralla et al., 2015). The filtered supernatant was harvested and lyophilized at a condensed temperature of −50°C at 110 millitorr chamber pressure for 48 h, and the obtained powder was weighed (Hossain et al., 2021; Montel Mendoza et al., 2014). An approximate 56 mg freeze‐dried sample from the culture supernatant was used in the next assay.

### 2.3 Determine the minimum inhibitory concentration (MIC) of freeze‐dried LAS

In this assay, the antibacterial activity of LAS against *S. enteritidis* was determined by a 96‐cell microplate through recording of the color change observed. This assay was described previously by the National Committee for Clinical Laboratory (CLSI) (Humphries et al., 2018), but it has been modified to determine *L. acidophilus* potential against *S. enteritidis*. In this method, 200 μL of BHI medium containing *S. enteritidis* (10^3^ CFU/mL) was dispensed in each well of columns 1–10, and the dilution concentration of LAS was achieved by double serial dilution by adding 200 μL of LAS in column 1. Afterward, a multichannel pipette was used to transfer and mix LAS from columns 1–10. The highest concentration incorporated into the plate is 5600 μg/mL, and the lowest achieved through serial dilution is 10 μg/mL. Columns 11 and 12 contained 200 μL of medium culture contaminated by *S. enteritidis* (10^3^ CFU/mL) and, 200 μL of LAS, which were considered as positive and negative controls, respectively. The microplate was incubated at 37°C for 72 h, and then a color change was observed. This test was performed in triplicate for this culture supernatant. The lowest concentration of LAS that inhibited the visible growth of a microorganism was considered as MIC.

### 2.4 Determination of population and microbial growth curve

In this stage, 100 μL of *S. enteritidis* (10^3^CFU/mL) was added in three sets of three microtubes containing 1 mL of UHT milk. MIC and sub‐MIC concentrations of LAS were added to the first and second microtube sets, and the third set was considered a positive control sample. All samples were incubated for 72 h at 8, 25, and 37°C. Every 8 h, 100 μL of the sample was added to the Xylose Lysine Deoxycholate (XLD ProMedia) medium, and after 24 h of incubation at 37°C, the colonies were counted.

A number of mathematical functions have been proposed that describe microbial growth curves. In this study, maximum generation time (MGT) and a specific growth rate (*μ*) have been presented.

Generation time, or doubling time is taken for cell division in an exponential phase, and the slope of this phase is described as a specific growth rate. There are several ways to express these concepts.

The calculation of specific growth rate and MGT is done according to the following mathematical equations (Maier & Pepper, 2015):

(1) $$\frac{dX}{dt}=\mathrm{\mu X}$$

where *X* is the number of mass of cells (mass/volume), *t* is time, and *μ* is the specific growth rate constant (1/time).

Rearrange:

(2) $$\frac{dX}{X}=\mu dt$$

Integrate:

(3) $$\int_{x0}^{X}\frac{dX}{X}=\mu \int_{0}^{t}dt$$

(4) $$\ln\;X=\mathrm{\mu t}+\ln\;X_{0}\;\text{or}\;X=X_{0}\;e^{\mathrm{\mu t}}$$

For *x* to be double:

(5) $$\frac{X}{X0}=2$$

Therefor:

(6) $$2=e^{\mathrm{\mu t}}$$

where *t*: generation time.

Likewise the MGT was obtained from:

(7) $$\;\mathrm{MGT}=\left(\frac{\log_{10}2}{\mu }\right)$$

### 2.5 Sensory evaluation

The treated samples were obtained by dissolving probiotic supernatant (5 × MIC) in 5 mL of UHT milk. Twenty untrained judges, consisting of thirteen males and seven female and smokers and non‐ smoker's aged 23–45 years were selected based on their ability to discriminate and reproduce the results.

All samples were served simultaneously to the panelists at refrigerator temperature (3–5°C) using paper cups labeled with 3‐digit codes from a random number table. Water was available for panel members to rinse their mouths between samples. Crackers were supplied as needed to remove flavor between tastings (Nogueras‐Iso et al., 2004).

After identifying odd samples, each panelist indicated the degree of difference between the odd and duplicated samples at the different levels: “none”, “slight”, “moderate”, and “extreme”, according to the method in ISSO6685:2017 (Lim et al., 2022). These qualitative data were converted to quantitative data as follows: “None – 0”, “Slight – 1”, “Moderate – 2”, and “Extreme – 3”.

In our study, the triangle test was used to determine the significant difference between the odd samples and other duplicate samples, and descriptive statistics were used to analyze the difference between the odd and duplicated samples.

### 2.6 Statistical analysis

The data were analyzed as mean ± standard error (SE). Results were analyzed by the Tukey's, tailed binomial, and one‐sample Kolmogorov–Smirnov test, with the help of IBM SPSS statistical 25 software program. Significant differences between means were defined at *p* < .05. Also, all experimental and statistical measurements were implemented in triplicate.

## 3 RESULTS

### 3.1 Determine the MIC of freeze‐dried LAS

Results from the antimicrobial activity of freeze‐dried LAS against *S. enteritidis* in the BHI medium are shown in Table 1. The experiment was conducted on a 96‐cell microplate at 37°C for 72 h. The MIC is the lowest concentration that prevents visible growth of *S. enteritidis* treated with freeze‐dried LAS, obtained at 1400 μg/mL, and the sub‐MIC of this antimicrobial agent that can induce stress and alter the expression of different bacterial genes is obtained at 700 μg/mL.

**TABLE 1:** Determination of the MIC and sub‐MIC by the 96‐well method of *S. enteritidis.*

Bacteria	Antimicrobial agent	MIC (μg mL^−1^)	Sub‐MIC (μg mL^−1^)
*S. enteritidis*	Freeze‐dried *LAS*	1400	700

### 3.2 Growth curves and metabolic parameters

Measurements of microbial growth curves and microbial parameters of studied microorganisms in inoculated UHT milk were calculated and measured by the mechanistic model by Baranyi and Roberts (Taoukis et al., 1999).

**FIGURE 1:**
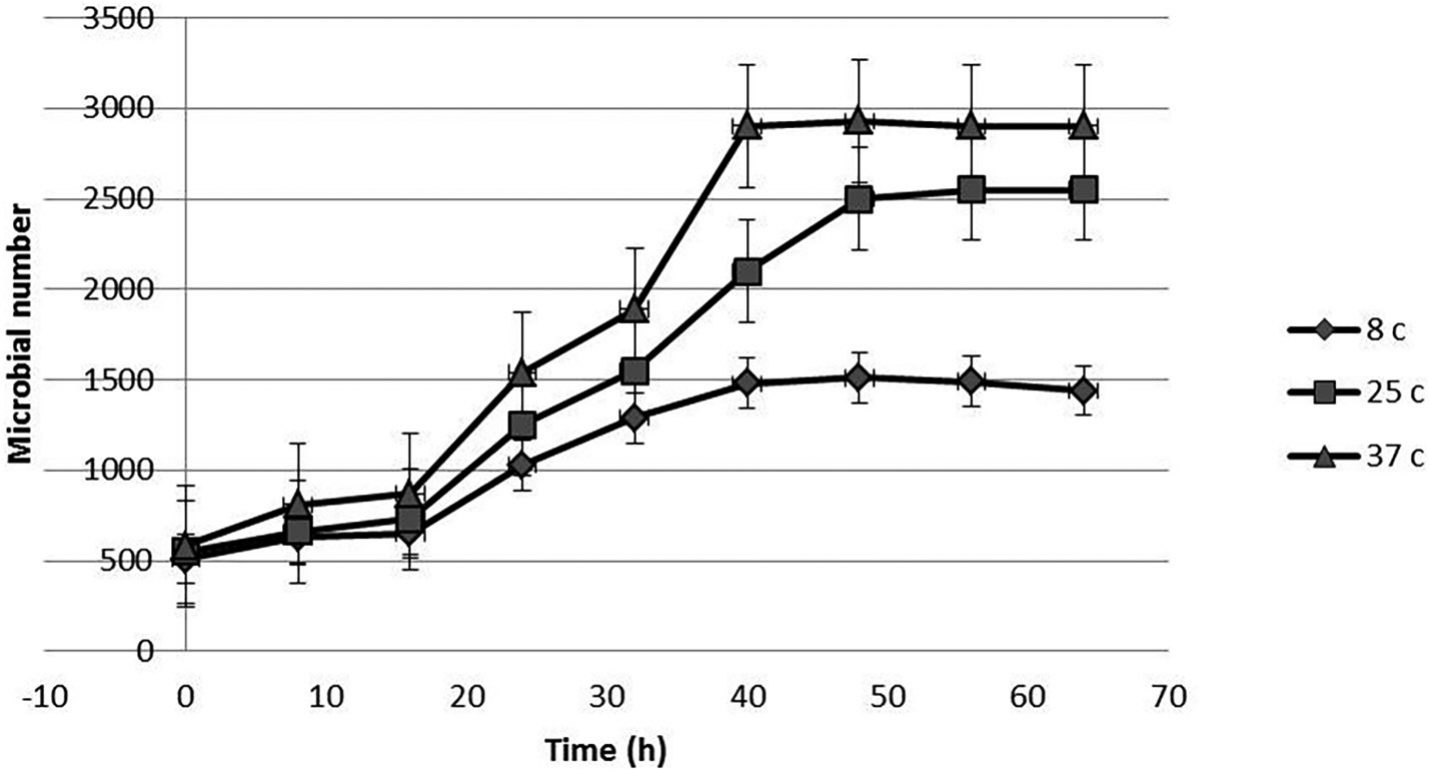
Growth curve of *S. entritidis* in zero concentration of freeze‐dried *LAS*.

**FIGURE 2:**
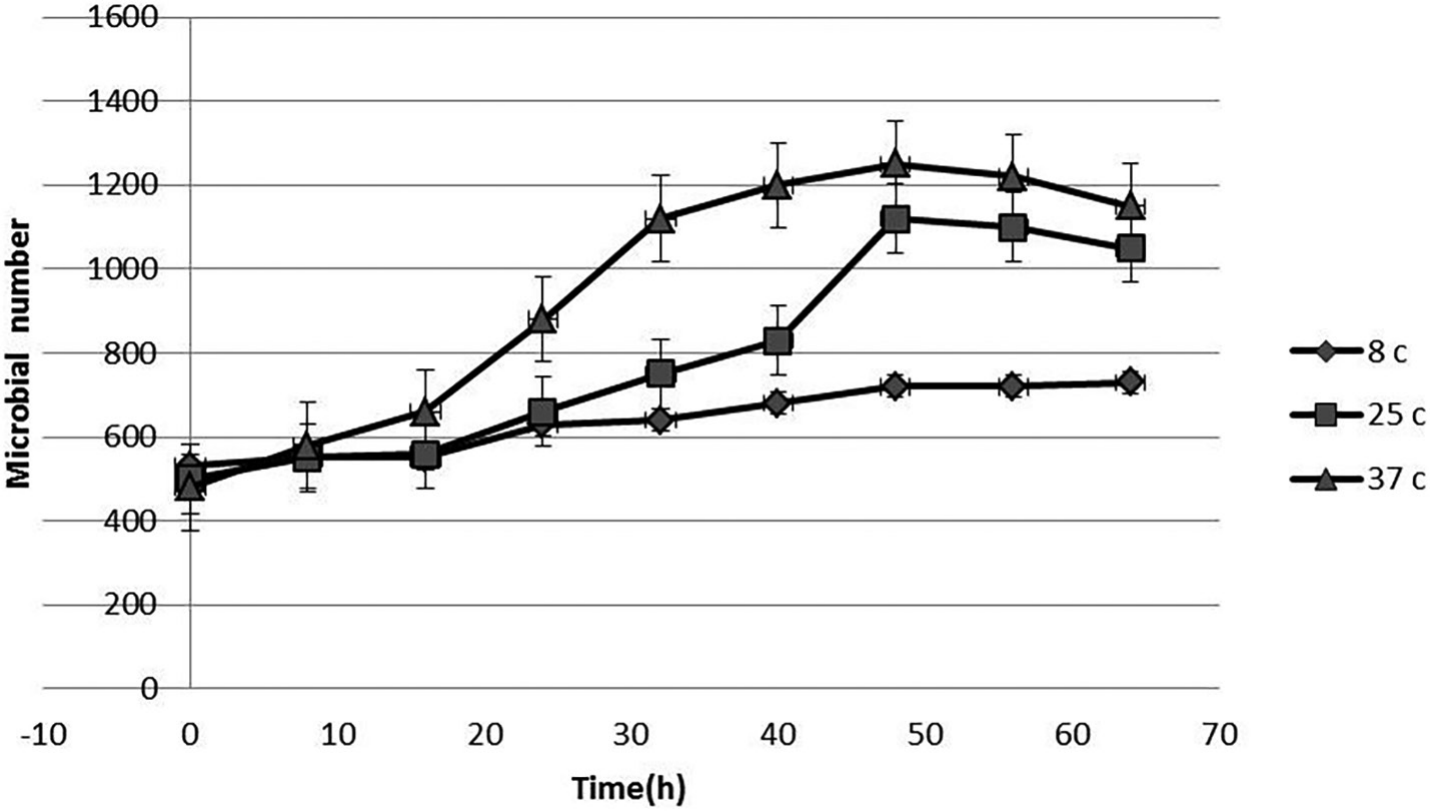
Growth curve of *S. enteritidis* in 700 μg/mL of freeze‐dried *LAS*.

**FIGURE 3:**
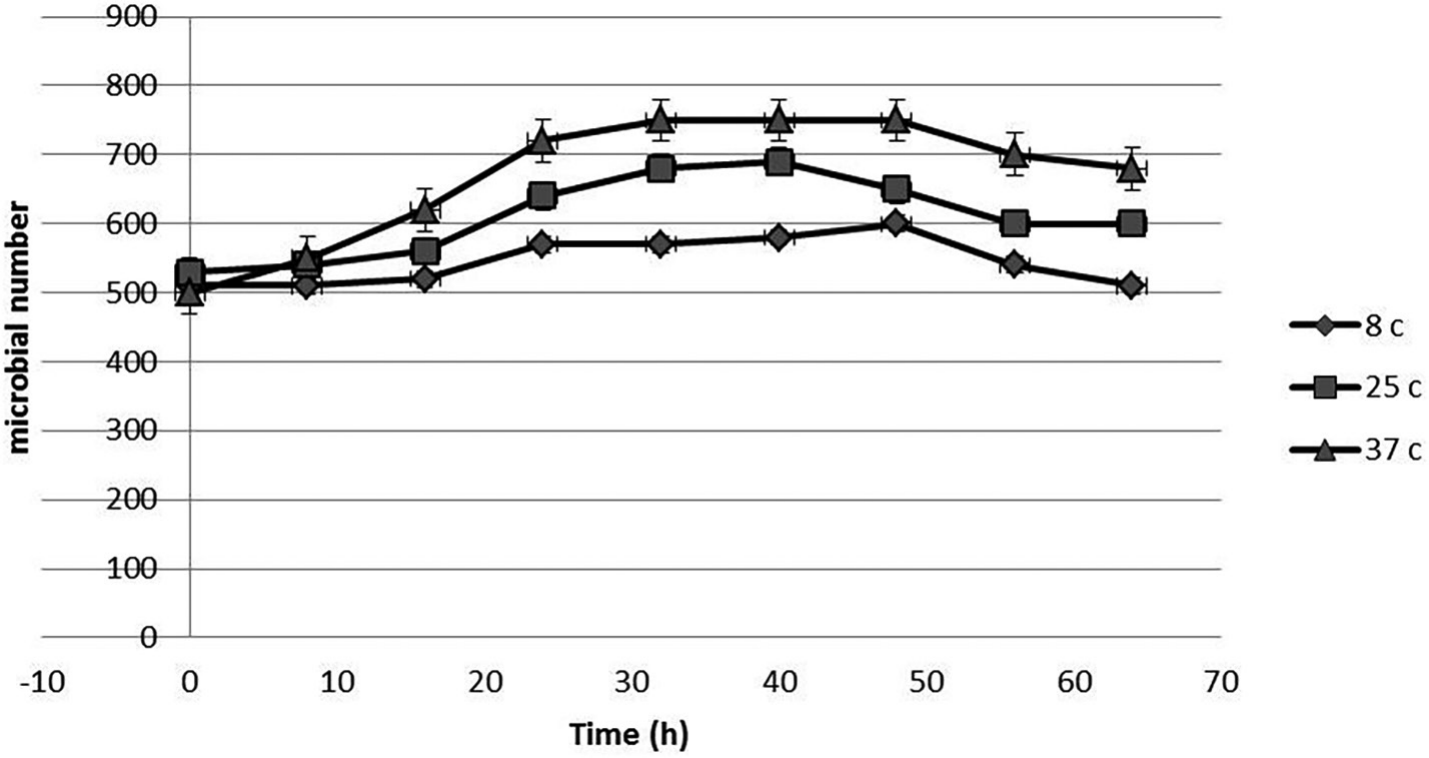
Growth curve of *S. enteritidis* in 1400 μg/mL of freeze‐dried *LAS*.

Incubation of *S. enteritidis* in UHT milk at constant temperatures provided typical growth curves. The effect of freeze‐dried LAS on the microbial growth curves of *S. enteritidis* is illustrated in Figures 1, 2, 3. Growth trials were performed in zero (positive control sample), MIC (1400 μg mL^−1^), and sub‐MIC (700 μg mL^−1^) concentrations of freeze‐dried LAS against *S. enteritidis* at 8, 25 and 37 ± 0.5°C. Microbial growth curves indicated that the exponential phase was started after 18 h, and in proportion to freeze‐dried LAS concentration and incubation temperatures, the microbial load has increased. At all temperatures, the lowest and highest amount of microbial load at the end of the exponential phase was related to the sample containing 1400 μg/mL freeze‐dried LAS and control samples, respectively, which indicated the antimicrobial effect of freeze‐dried LAS.

Maximum specific growth rate and MGT of *S. enteritidis* in UHT milk at 8, 25 and 37 ± 0.5°C and in zero (positive control sample), MIC (1400 μg mL^−1^) and sub‐MIC (700 μg mL^−1^) concentrations of freeze‐dried LAS against *S. enteritidis* are summarized in Tables 2 and 3. These were calculated by the slope of the linear part of the microbial number vs. t curve. The inverse relationship between *μ*_max_ and MGT is declared in the presented parameters. *μ*_max_ increased from 0.0059 h^−1^ at 8°C and 1400 μg/mL freeze‐dried LAS to 0.055 h^−1^ in 37°C and zero concentration of freeze‐dried LAS; on the other hand, MGT decreased from 51.01 h at 8°C and 1400 μg/mL freeze‐dried LAS to 5.47 h at 37°C and zero concentration of freeze‐dried LAS.

**TABLE 2:** Maximum Specific grow rate (h^−1^) of *S. enteritidis* in different concentrations of freeze‐dried LAS.

Temperature (°C)	Concentration (μg mL^−1^)		
	Zero	700	1400
8	0.034 a	0.0062 b	0.0059 b
25	0.038 c	0.021 d	0.0087 e
37	0.055 f	0.033 g	0.015 h

*Note*: Different letters denote significant differences (*p* < .05).

**TABLE 3:** Maximum Generation Time (h) of *S. enteritidis* in different concentrations of freeze‐dried LAS.

Temperature (°C)	Concentration (μg mL^−1^)		
	Zero	700	1400
8	8.85 a	48.16 b	51.01 c
25	7.92 d	14.33 e	34.59 f
37	5.47 g	9.12 h	20.06 i

*Note*: Different letters denote significant differences (*p* < .05).

### 3.3 Sensory evaluation

These sensory evaluations conducted by the triangle method and analyzed by a one‐tailed binomial comparison test are shown in Table 4. According to the triangle test, 36% (11 out of 30) of the participants correctly recognized the target sample, and there were no significant differences between any treatment samples and other ones (*p* > .05). This test requires at least 15 correct answers to confirm that the participants have recognized a significant difference between the samples (Lawless & Heymann, 2010). Therefore, these results demonstrate that freeze‐dried LAS utilization in UHT milk, as an increasing shelf‐life factor, will not affect a change in sensory characteristics.

**TABLE 4:** Results of sensory evaluation (triangle method).

Test	Data
Number of panelists	30
Number of correct response	11
Number of not correct responses	19
Probability of correct answers	0.36
Guessing probability	0.33
Alpha	0.05

**FIGURE 4:**
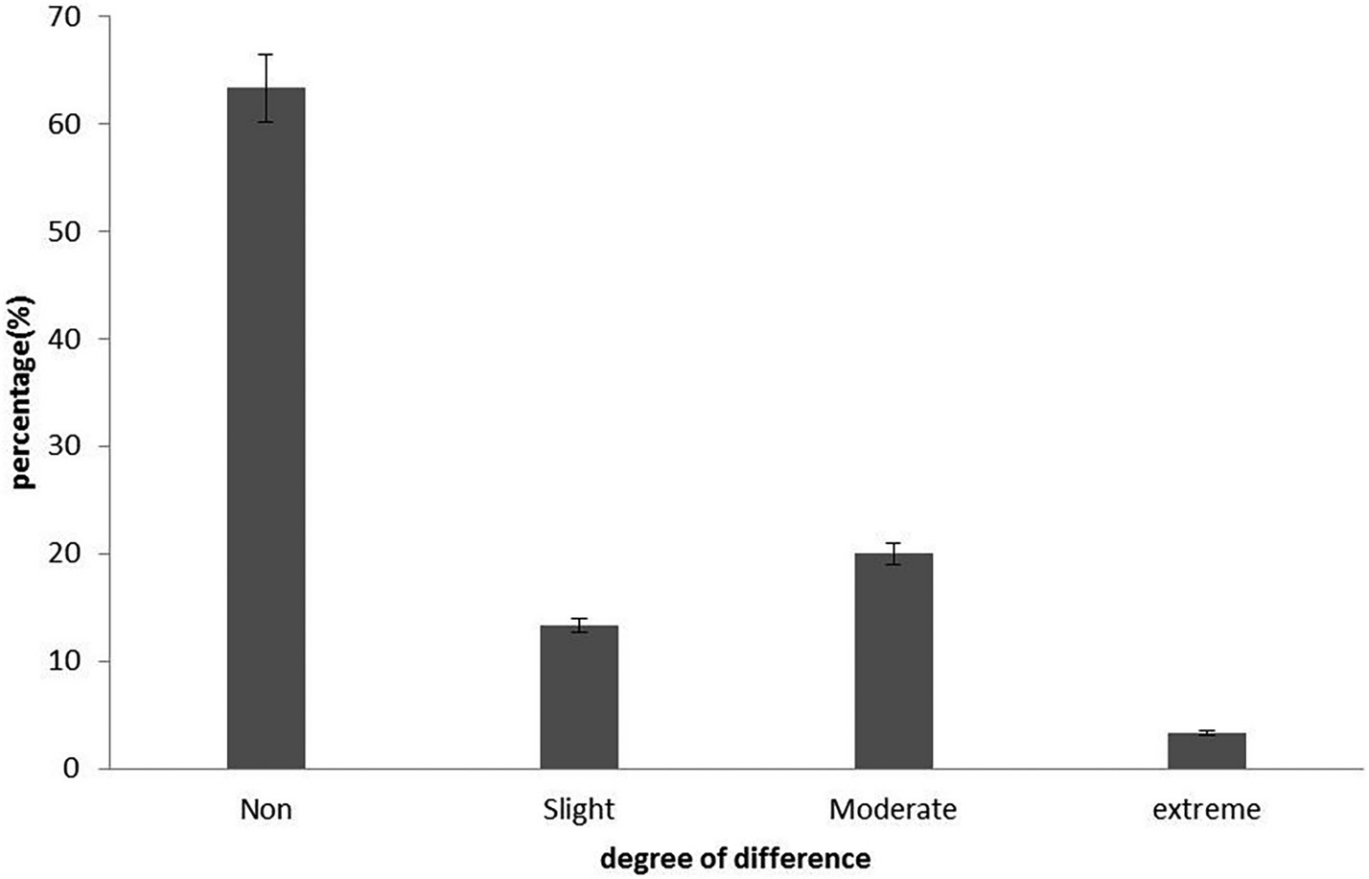
Selection of degree of difference “None”, “Slight”, “Moderate”, “Much”, and “Extreme” from the correct responses.

Among the samples that were correctly recognized, the degree of difference with the other two samples is shown in Figure 4. Four panelists (13.3%) selected “slight”, six panelists (20%) selected “moderate”, and only one panelist (3.3%) selected “extreme. It should be pointed out that the number of not‐correct samples “(None)” was observed to be significantly (*p* < .05) higher than in other groups. Descriptive statistics analysis to summarize the responses of different levels between samples is shown in Table 5.

**TABLE 5:** Analysis of the triangle data.

Mean	0.6
Standard error	0.169
Standard deviation	0.927
Variance	0.861
Sample variance	1.062
Range	3
Sum	19
Count	30

## 4 DISCUSSION

Although there are many methods to prevent the growth of pathogens in the food industry, probiotics are being used as a new method for improving food safety and enhancing food shelf life. They have been used to replace conventional methods of food preservation, such as antibiotic therapy, that increase the resistance of pathogens to drugs. Beneficial effects of probiotics include: (a) anti‐pathogenic activity, (b) immunomodulation activity, (c) enhancing the nutritional value of food products, (d) improving flavor and texture of the final product, (e) anti‐diabetic activity, (f) controlling and reducing serum cholesterol, (g) preventing gastrointestinal infection, and (h) reducing lactose intolerance symptoms, to cause utilization of these microorganisms in functional foods that may contain one or more selected microbial strains (Kerry et al., 2018).

Several attempts have proven the ability of probiotics and their metabolites (postbiotics) to inhibit pathogenic microorganisms related to the production of antimicrobial agents (Pakbin et al., 2023; Salleh et al., 2014).

In our study, we determined the MIC of *S. enteritidis* treated with freeze‐dried LAS by 96‐cell microplate; likewise, many researchers suggested this method for the determination of microbial MIC in confront with the CFS of probiotic species (Ji & Yang, 2021; Moghadam et al., 2021).

**FIGURE 5:**
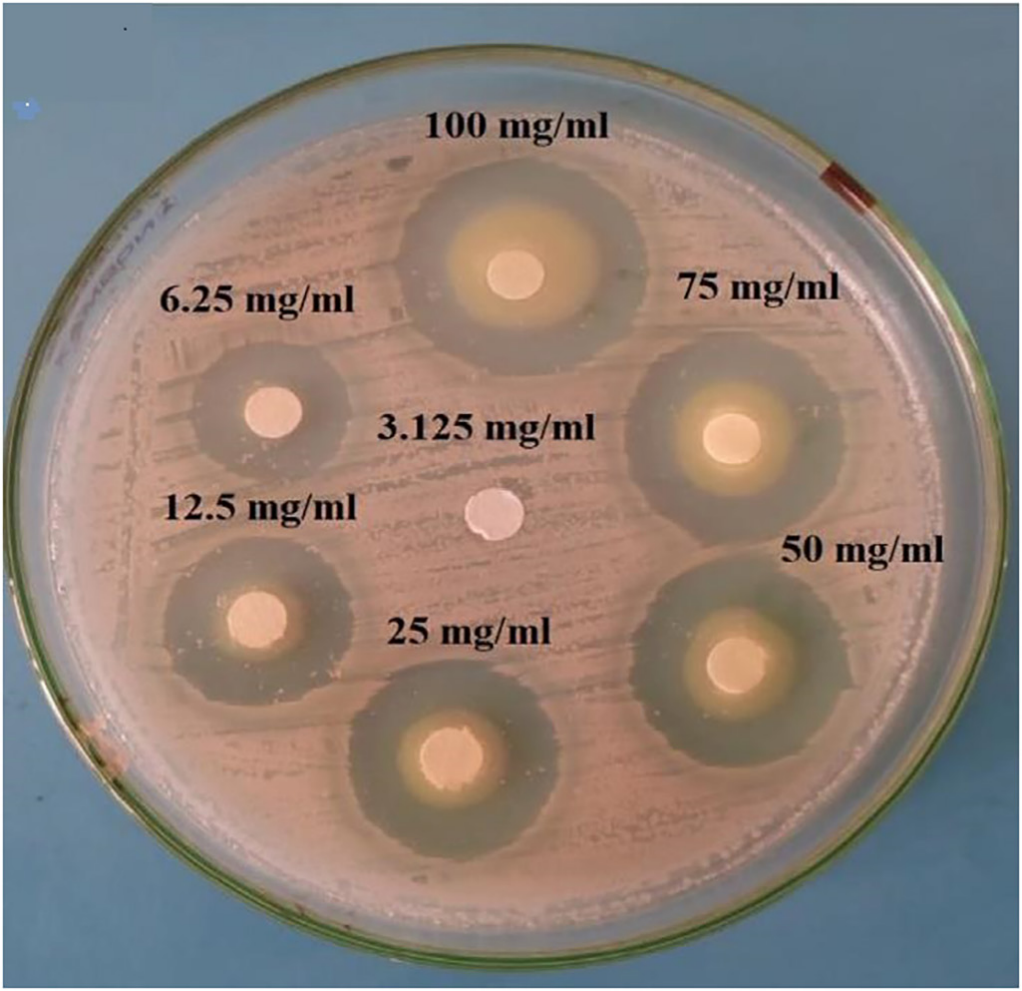
Minimum inhibitory concentration of *Lactobacillius rhamnosus* EMCC 1105 supernatant against *Clostridium perfringens* (6.25 mg/mL).

Because *L. acidophilus* is a crucial commercial starter strain, it is necessary to overcome the limitations of using this probiotic in the dairy industry. This bacterium has many health‐giving properties of other probiotic bacteria; therefore, it is used for fermentation along with other starters such as *Streptococcus thermophiles* and *Lactobacillus delbrueckii* ssp that are growing faster than *L. acidophilus* during fermentation but unable to survive in the passage of the human gastrointestinal tract (Ng et al., 2011). The use of LAS as a postbiotic, while removing the limitation of the low growth rate of this bacterium in milk, also brings all the health‐giving features and makes food durable (Ji & Yang, 2021). The antimicrobial activity of postbiotics obtained in the present study has also been reported previously for other probiotic supernatants. The MIC of Staphylococcus strains isolated from milk treated with *Bifidobacterium bifidum* and *Lactococcus lactis* probiotic supernatant was reported at 125 μg/mL by Moghadam et al (Moghadam et al., 2021). In the same study, Gamal M. Hamad et al. surveyed the anti‐*Clostridium perfringens* effect of four probiotic strain supernatants in Egyptian poultry, and they showed that *Lactobacillus rhamnosous* EMCC 1105 has a higher anti‐clostridia potential (Hamad et al., 2020). In this paper, the MIC was achieved at 6.25 mg/mL, and the inhibition zone diameter at a concentration of 100 mg/mL was calculated at 30 mm, as shown in Figure 5.

According to our study, the simultaneous use of different food storage methods, which is known as hurdle technology, has a significant effect on increasing the shelf life of food. Meanwhile, the simultaneous control of temperature and the use of preservative agents can have a significant impact on preventing the growth of pathogenic microorganisms (Leistner & Gorris, 1995). This is the first report to analyze the combination of temperature and probiotic supernatant treatment on a microbial growth curve. This study, a survey of the growth pattern of *S. enteritidis* in the vicinity of freeze‐dried LAS, shows that the growth of this pathogen was prevented. Few reports have been done on the joining between applying probiotic supernatant and the decrease in maximum specific growth rate. In the study conducted by Anand Kumar et al. the specific growth rate of *Salmonella enteric* serovar Typhi alone and the vicinity of *Lactococcus lactic* (MTCC‐440) was reported as 0.695 h^−1^ and 0.35 h^−1^, respectively (Kumar et al., 2018). In previous studies, HPLC analysis confirmed the presence of lactic, acetic, and citric acids in the probiotic supernatant. The inhibitory effect of these compounds is caused by their penetration inside the cell membrane and as a result of the reduction of PH and disruption of the membrane potential.

In comparison with Figures 1, 2, 3, as can be seen in Figure 3, due to the presence of a high level of freeze‐dried LAS, the exponential phase is not sharply present. The parameters obtained for *μ*_max_ and MGT were consistent with the visual analysis of microbial growth curves. As can be seen in Tables 1, 2, 3, the higher value of *μ*_max_ for *S. enteritidis* was observed at 37°C and zero concentrations of freeze‐dried LAS, and a higher value of MGT for this microorganism was observed at 8°C and 1400 μg/mL of freeze‐dried LAS. In this paper, the simultaneous effect of incubation temperature and an antimicrobial agent on inhibiting microbial growth is clearly visible. The results for this parameter were close to those obtained by Hao Line et al. (Lin et al., 2016), which predicted the growth of *Pseudomonas fluorescens* in milk. In this study, the maximum growth rates for storage at 4, 15, and 29°C were 0.056, 0.17, and 0.46 h^−1^, respectively, and the MGT was 6.42, 2.82, and 0.81 h, respectively. Other studies have also reported a significant effect of temperature on microbial growth parameters. Zuzana et al. evaluated the growth of *Lactobacillus plantarum* in milk in dependence on temperature and reported a significant effect of temperature on a specific growth rate. These authors reported that by increasing the incubation temperature from 8°C to 37°C, the microbial specific growth rate (*μ*) increased from 0.001 to 0.744 h^−1^ (Matejčeková et al., 2016).

The results of the sensory analysis (triangle test) of newly processed UHT milk by adding freeze‐dried LAS are shown in Table 4. There were no significant (*p* > .05) differences between odd and non‐treatment samples. These results demonstrate that fortifying UHT milk with freeze‐dried LAS will not result in a change in sensory characteristics and perceptible of odd samples.

## 5 CONCLUSIONS

The results of this study showed that by adding freeze‐dried LAS as an antimicrobial agent to UHT milk contaminated with *S. enteritidis*, the growth rate of this pathogen can be reduced according to the incubation temperature. On the other hand, sensory evolution by triangle test demonstrated that there was no significant difference between the new formulation of UHT milk and control samples. Since no side effects have been reported from the metabolites of probiotics (postbiotics), they can be considered as suitable substitutes for food additives with known side effects. While postbiotics increase the shelf life of foods, they can also have the anti‐pathogenic benefits of probiotics.

## AUTHOR CONTRIBUTIONS

**Abbas Kamali:** Formal analysis (equal); methodology (equal); resources (equal); software (equal); validation (equal); writing – original draft (equal). **Hedayat Hosseini:** Formal analysis (equal); funding acquisition (equal); investigation (equal); resources (equal); supervision (equal). **Razzagh Mahmoudi:** Project administration (equal); resources (equal); software (equal); supervision (equal). **Babak Pakbin:** Conceptualization (equal); data curation (equal); software (equal); writing – review and editing (equal). **Nematollah Gheibi:** Formal analysis (equal); investigation (equal); methodology (equal). **Amir Mohammad Mortazavian:** Data curation (equal); formal analysis (equal); resources (equal); validation (equal). **Saeideh Shojaei:** Software (equal); validation (equal); visualization (equal).

## CONFLICT OF INTEREST STATEMENT

All the authors have declared no conflicts of interest.

## Data Availability

We confirm that all the data and findings of this study are available within the article.
